# Epidemiology of influenza in Ethiopia: findings from influenza sentinel surveillance and respiratory infection outbreak investigations, 2009–2015

**DOI:** 10.1186/s12879-018-3365-5

**Published:** 2018-09-03

**Authors:** Abyot Bekele Woyessa, Mesfin Mengesha, Desalegn Belay, Adamu Tayachew, Workenesh Ayele, Berhane Beyene, Woubayehu Kassa, Etsehiwot Zemelak, Gelila Demissie, Berhanu Amare, Lucy Boulanger, Carolina Granados, Thelma Williams, Israel Tareke, Soatiana Rajatonirina, Daddi Jima

**Affiliations:** 1 Center for Public Health Emergency Management, Ethiopian Public Health Institute, PO BOX 1242, Addis Ababa, Ethiopia; 2 CDC, Addis Ababa, Ethiopia; 3 CDC, Atlanta, USA; 4 WHO, Addis Ababa, Ethiopia; 5 WHO, AFRO, Brazzaville, Congo

**Keywords:** Influenza, Surveillance, Outbreak, Epidemiology, Virus, Laboratory, Ethiopia

## Abstract

**Background:**

Influenza is an acute viral disease of the respiratory tract which is characterized by fever, headache, myalgia, prostration, coryza, sore throat and cough. Globally, an estimated 3 to 5 million cases of severe influenza illness and 291 243–645 832 seasonal influenza-associated respiratory deaths occur annually. Although recent efforts from some African countries to describe burden of influenza disease and seasonality, these data are missing for the vast majority, including Ethiopia. Ethiopia established influenza sentinel surveillance in 2008 aiming to determine influenza strains circulating in the country and know characteristics, trend and burden of influenza viruses.

**Methods:**

We used influenza data from sentinel surveillance sites and respiratory disease outbreak investigations from 2009 to 2015 for this analysis. We obtained the data by monitoring patients with influenza-like illness (ILI) at three health-centers, severe acute respiratory infection (SARI) at five hospitals and investigating patients during different respiratory infection outbreaks. Throat-swab specimens in viral transport media were transported to the national reference laboratory within 72 h of collection using a cold-chain system. We extracted viral RNA from throat-swabs and subjected to real-time PCR amplification. We further subtyped and characterized Influenza A-positive specimens using CDC real-time reverse transcription PCR protocol.

**Results:**

A total of 4962 throat-swab samples were collected and 4799 (96.7%) of them were tested. Among them 988 (20.6%) were influenza-positive and of which 349 (35.3%) were seasonal influenza A(H3N2), 321 (32.5%) influenza A(H1N1)pdm2009 and 318 (32.0%) influenza B. Positivity rate was 29.5% in persons 5–14 years followed by 26.4% in 15–44 years, 21.2% in > 44 years and 6.4% in under five children. The highest positivity rate observed in November (37.5%) followed by March (27.6%), December (26.4%), October (24.4%) and January (24.3%) while the lowest positivity rate was in August (7.7%).

**Conclusion:**

In Ethiopia, seasonal Influenza A(H3N2), Influenza A(H1N1)pdm2009 and Influenza B viruses were circulating during 2009–2015. Positivity rate and number of cases peaked in November and December. Influenza is one of public health problems in Ethiopia and the need to introduce influenza vaccine and antivirus is important to prevent and treat the disease in future.

## Background

Influenza viruses are enveloped viruses of the family *Orthomyxoviridae* that contain a segmented RNA genome. Influenza is an acute viral disease of the respiratory tract which is characterized by some or all of the following sign and symptoms: fever, headache, myalgia, prostration, coryza, sore-throat and cough [1]. Of the three types of influenza viruses, A, B, and C, the first two are associated with significant seasonal morbidity and mortality [2, 3]. Influenza A is more prevalent and leads to greater mortality in humans than influenza B [4, 5].Type A influenza has subtypes that are determined by the surface antigens hemagglutinin (H) and neuraminidase (N) [6].

Globally, an estimated 3 to 5 million cases of severe influenza illness [7] and 291 243–645 832 seasonal influenza-associated respiratory deaths occur annually [8]. In Africa, influenza-related incidence data remain inadequate and outcome data are still very limited despite of some surveillance information [9].

In temperate regions, seasonal influenza typically occurs every year in the late fall or winter [10] while the seasonality is less clearly defined in tropical and subtropical regions [11]. Seasonality patterns of influenza in eastern Africa including Ethiopia, have not been clearly established [12]. However, some evidences from different studies indicated that Influenza A (H1N1)pdm2009, Seasonal Influenza A(H3N2) and Influenza B are circulating in different countries of sub-Saharan Africa [13–15].

To address this gap and generate evidence in Ethiopia, severe acute respiratory infections (SARI) and influenza like illness (ILI) sentinel surveillance was established in November 2008 [16]. The Ethiopian Public Health Institute (EPHI) has adapted and prepared an influenza sentinel surveillance implementation manual [17]. The establishment of influenza sentinel surveillance is aimed to detect new influenza strains capable of or having the potential to cause a pandemic, determine the characteristics of influenza, characterize and monitor trends in illnesses and deaths attributable to severe acute respiratory infections, determine the proportions of confirmed cases of influenza among SARI in-patients and/or among ILI out-patients and finally to provide information on the contribution of influenza to the burden of respiratory diseases in order to prioritize resources and plan appropriate public health interventions in Ethiopia.

## Methods

### Sentinel surveillance sites and time

The ILI program is a health center-based sentinel surveillance system that monitors children and adults presenting with sudden onset of fever > 38 °C and cough or sore throat. The first ILI sentinel site was established at Shiromeda Health Center (HC) in Addis Ababa in November 2008. In October 2010 two more ILI sentinel sites, Kolfe and Akaki HCs, were established in other parts of Addis Ababa. The SARI program is a hospital-based sentinel surveillance system that monitors children and adults hospitalized with pneumonia across five hospitals in Ethiopia. In November 2008, SARI surveillance was established and implemented in Yekatit 12 Memorial Hospital in Addis Ababa. In October 2013, four additional SARI sentinel surveillance sites were introduced at Adama Teaching Hospital in Oromia regional state, Adare Hospital in Southern Nations, Nationalities and Peoples’ (SNNP) regional state, Felegehiwot Hospital in Amhara regional state and Mekele Hospital in Tigray regional state. In this study we have included the findings from January 1, 2009 to December 31, 2015.

### Case definitions and data collection

ILI was defined as any person presenting with sudden onset of fever ≥38 °C (axillary measured) AND cough or sore throat in the absence of other diagnosis [17, 18]. SARI was defined as any person> 5 years presenting with symptoms of acute lower respiratory infection with sudden onset of fever ≥38 °C, AND cough or sore throat, AND shortness of breath or difficulty breathing, AND requiring hospitalization [17, 18] or any child ≤5 years that fulfilled the case definition of pneumonia and severe pneumonia as per the Integrated Management of Childhood Illnesses protocol [19].

Patients were enrolled in this surveillance program during their visit to pre designated HCs for ILI sentinel surveillance, or to hospitals for SARI sentinel surveillance. At the ILI sentinel surveillance sites throat swab samples were systematically collected from the first five ambulatory patients of all ages who fulfilled the case definition for ILI per week. Similarly throat swab samples were also collected from all patients who fulfilled the SARI case definition and admitted to hospitals designated as SARI sentinel surveillance sites. In addition throat swab samples were collected by investigating teams during reported respiratory infection outbreaks. Specimen were collected within seven days after the first onset of symptoms. Patients’ demographic information including age, sex and place of residence were recorded on a standard reporting format. Throat swabs samples were placed in viral transport media (VTM) and stored at 4 °C until they were transported to the National Influenza Laboratory (NIL) at EPHI twice a week for laboratory analysis. We transported throat swab specimens in viral transport media to the NIL at EPHI within 72 h of collection using a cold chain system.

### Laboratory methods

We extracted viral RNA from throat swabs and subjected to real-time PCR amplification with parameters set for influenza testing, according to Center for disease Control and Prevention (CDC) protocol using reagents obtained from Influenza Reagent Resources (IRR). We further sub-typed and characterized influenza A-positive specimens using the CDC real-time reverse transcription PCR protocol [20].

### Analysis

We extracted influenza sentinel surveillance data from January 1, 2009 to December 31, 2015 from the national flu database. The data was categorized by laboratory results, age groups, sex and place. We entered and cleaned data by Epi info version 3.5.2 and calculated proportions using Microsoft excel. We assessed influenza seasonality by evaluating the months having peak and trough proportions of influenza positive specimens each year.

## Results

**Fig. 1:**
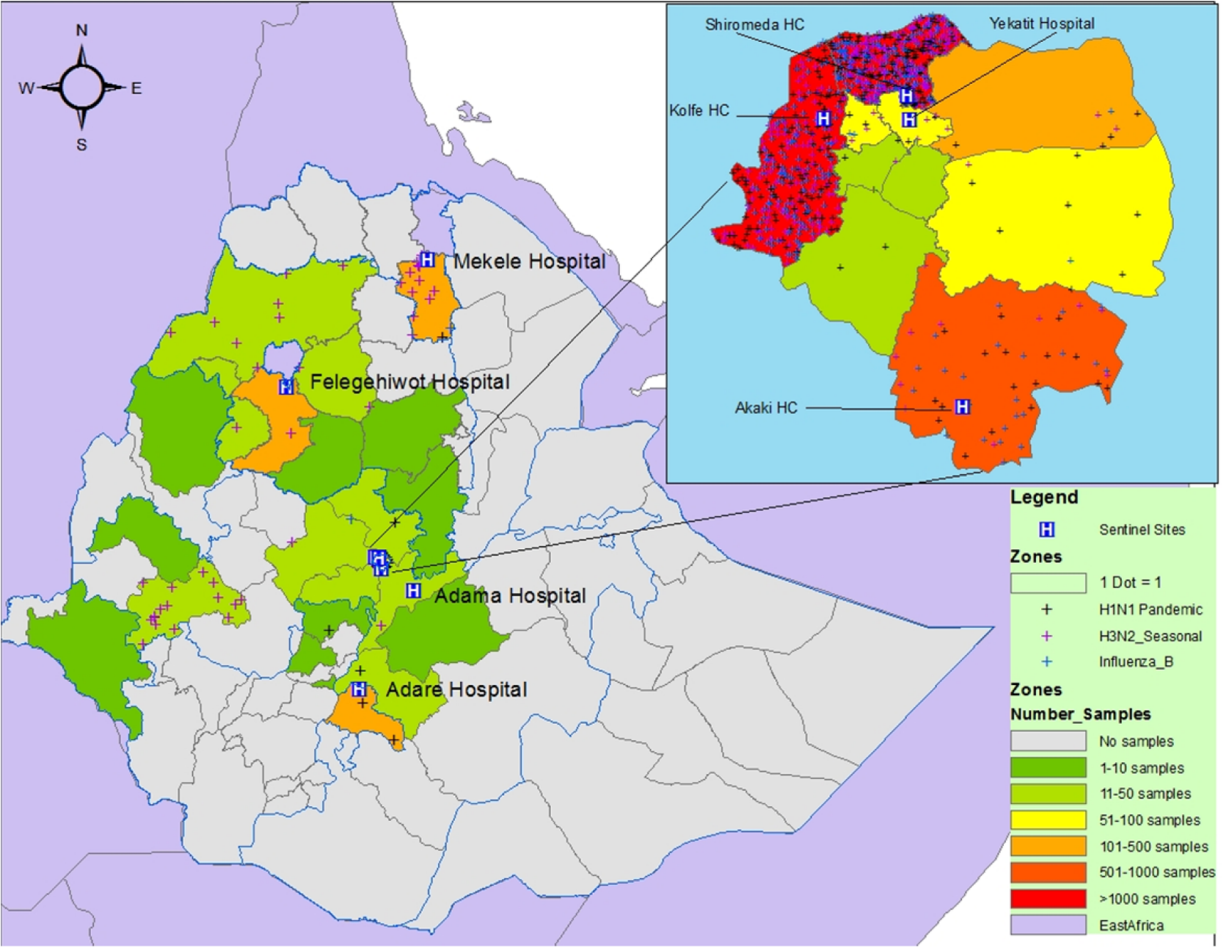
Map showing sentinel sites, areas from where samples were collected and influenza positivity by subtypes by zone

From January 1, 2009 through December 31, 2015, a total of 4962 patients which fulfilled ILI and SARI case definitions were enrolled and throat samples collected at designated influenza sentinel site health facilities and from respiratory infection outbreak investigations in different parts of the country (Fig. 1). The mean age of the sampled cases was 15 years (Range: 1 month to 82 years) and the median age was 11 years (Inter quartile range 2–25 years). Of the total, 4799 (96.7%) were tested for influenza by RT-PCR and 988 (20.6%) were found to be positive for different influenza viruses. Among the positive cases 349 (35.3%) were caused by seasonal influenza A (H3N2), 321(32.5%) by influenza A (H1N1) pdm2009 and the remaining 318 (32%) by Influenza B. The mean age of the influenza positive cases was 19 years (Range: 2 months to 72 years) and the median age was 17 years (Inter quartile range 8–26 years). The positivity rate was 5% in persons under 2 years, 10.3% in 2–4 years, 29.5% in 5–14 years, 26.3 in 15–49 years, 21.1 in 50–64 years and 16.7% in person greater than 64 years (Table 3). Of the total positive cases 568 (57.5%) were females with a positivity rate of 21.3% and 420 (42.5%) were males with a positivity rate of 19.7%. Among total 4962 specimens collected, 3369 (67.9%) were ILI and 1381 (27.8%) were SARI cases while the remaining 212 (4.3%) were sampled during different respiratory disease outbreaks.

### Influenza like illness (ILI)

From 3369 ILI specimens collected overtimes, 3323 (99%) were tested and of them 891 (27%) were turned positive for different influenza strains. Among the ILI specimens positive for Influenza 46 (79%), 10 (17%) and 2 (3%) were attributed to Influenza A(H1N1)pdm2009, A(H3N2) and Influenza B respectively. The positive influenza cases were unevenly distributed among different age groups. The mean age of the influenza positive cases was 19 years (Range: 2 months to 72 years) and the median age was 17 years (Inter quartile range 8–25 years). Among the total positive cases of ILI specimens, 3% were among under 2 years, 5% were among 2–4 years, 37% among 5–14 years, 51% were among 15–49 years old, 4% from 50 to 64 years and 1% among greater than 64 years (Table 1).

**Table 1:** Influenza activity (positivity, circulating types and subtypes) among patients seen as outpatients and presenting with ILI in Ethiopia, 2009–2015

Characteristic	Samples Collected	Samples Tested	Influenza Positive	Influenza types and subtypes			
				A	A(H1N1) pdm2009	A(H3N2)	B
	*N*	*N* (%) ^a^	*N* (%)^b^	*N* (%)^c^	*N* (%)^c^	*N* (%)^c^	*N* (%)^c^
All cases	3369	3323 (99)	891 (27)	579 (65)	264 (30)	315 (35)	312 (35)
Year							
2009	64	40 (63)	8 (20)	6 (75)	2 (25)	4 (50)	2 (25)
2010	80	80 (100)	4 (5)	4 (100)	4 (100)	0 (0)	0 (0)
2011	61	61 (100)	19 (31)	8 (42)	2 (11)	6 (32)	11 (58)
2012	797	792 (99)	211 (27)	174 (82)	160 (76)	14 (7)	37 (18)
2013	1047	1043 (100)	347 (33)	214 (62)	22 (6)	192 (55)	133 (38)
2014	797	792 (99)	228 (29)	132 (58)	41 (18)	91 (40)	96 (42)
2015	523	515 (98)	74 (14)	41 (55)	33 (45)	8 (11)	33 (45)
Age							
< 2	191	186 (97)	23 (12)	18 (78)	10 (43)	8 (35)	5 (22)
2–4	228	222 (97)	41 (18)	26 (63)	16 (39)	10 (24)	15 (37)
5–14	1013	997 (98)	331 (33)	198 (60)	84 (25)	114 (34)	133 (40)
15–49	1720	1705 (99)	453 (27)	312 (69)	147 (32)	165 (36)	141 (31)
50–64	153	149 (97)	34 (23)	20 (59)	6 (18)	14 (41)	14 (41)
> =65	48	48 (100)	9 (19)	5 (56)	1 (11)	4 (44)	4 (44)
Missed	16	16 (100)	0 (0)	0 (0)	0 (0)	0 (0)	0 (0)
Sex							
Female	2025	1993 (98)	521 (26)	325 (62)	147 (28)	178 (34)	196 (38)
Male	1344	1330 (99)	370 (28)	254 (69)	117 (32)	137 (37)	116 (31)
Site							
Akaki HC	244	242 (99)	53 (22)	30 (57)	18 (34)	12 (23)	23 (43)
Kolfe HC	1366	1360 (100)	344 (25)	216 (63)	103 (30)	113 (33)	128 (37)
Shiromeda HC	1759	1721 (98)	494 (29)	333 (67)	143 (29)	190 (38)	161 (33)

^a^Denominator is the number who were sampled

^b^Denominator is the number who were tested

^c^Denominator is the number who tested positive

### Severe acute respiratory infections (SARI)

From 1381 SARI specimens collected overtimes, 1268 (92%) were tested and of them 39 (3%) were turned positive for different influenza strains. Among the SARI specimens positive for Influenza 11 (28%), 24 (62%) and 4 (10%) were attributed to Influenza A(H1N1)pdm2009, A(H3N2) and Influenza B respectively. The mean age of the influenza positive cases was 4.7 years (Range: 2 months to 50 years) and the median age was 1 year (Inter quartile range 2.3 years). Among the total positive cases of SARI specimens, 69% were among under 2 years, 15% were among 2–4 years, 8% among 5–14 years, 5% were among 15–49 years old and 3% from 50 to 64 years old (Table 2).

**Table 2:** Influenza activity (positivity, circulating types and subtypes) among patients hospitalized with SARI in Ethiopia, 2009–2015

Characteristic	Samples Collected	Samples Tested	Influenza Positive	Influenza types and subtypes			
				A	A(H1N1) pdm2009	A(H3N2)	B
	*N*	*N* (%)^a^	*N* (%)^b^	*N* (%)^c^	*N* (%)^c^	*N* (%)^c^	*N* (%)^c^
All cases	1381	1268 (92)	39 (3)	35 (90)	11 (28)	24 (62)	4 (10)
Year							
2009	142	52 (37)	3 (6)	3 (100)	0 (00)	3 (100)	0 (0)
2010	74	74 (100)	2 (3)	2 (100)	2 (100)	0 (0)	0 (0)
2011	53	53 (100)	1 (2)	1 (100)	0 (0)	1 (100)	0 (0)
2012	62	62 (100)	0 (0)	0 (0)	0 (0)	0 (0)	0 (0)
2013	159	138 (100)	12 (9)	10 (83)	0 (0)	10 (83)	2 (17)
2014	609	608 (100)	17 (3)	15 (88)	7 (41)	8 (47)	2 (12)
2015	282	281 (100)	4 (1)	4 (100)	2 (50)	2 (50)	0 (0)
Age							
< 2	885	805 (91)	27 (3)	23 (85)	7 (26)	16 (59)	4 (15)
2–4	258	238 (92)	6 (3)	6 (100)	1 (17)	5 (83)	0 (0)
5–14	146	135 (92)	3 (2)	3 (100)	2 (67)	1 (33)	0 (0)
15–49	55	53 (96)	2 (4)	2 (100)	1 (50)	1 (50)	0 (0)
50–64	12	12 (100)	1 (8)	1 (100)	0 (0)	1 (100)	0 (0)
> =65	8	8 (100)	0 (0)	0 (0)	0 (0)	0 (0)	0 (0)
Missed	17	17 (100)	0 (0)	0 (0)	0 (0)	0 (0)	0 (0)
Sex							
Female	617	570 (92)	17 (3)	14 (82)	5 (29)	9 (53)	3 (18)
Male	764	698 (91)	22 (3)	21 (95)	6 (27)	15 (68)	1 (5)
Site							
Adama Hospital	47	47 (100)	1 (2)	1 (100)	0 (0)	1 (100)	0 (0)
Adere Hospital	127	123 (97)	4 (3)	3 (75)	3 (75)	0 (0)	1 (25)
Felegehiwot Hospital	259	259 (100)	4 (2)	4 (100)	0 (0)	4 (100)	0 (0)
Mekele Hospital	306	304 (99)	14 (5)	13 (93)	1 (7)	12 (86)	1 (7)
Yekatit 12 Hospital	642	535 (83)	16 (3)	14 (88)	7 (44)	7 (44)	2 (13)

^a^Denominator is the number who were sampled

^b^Denominator is the number who were tested

^c^Denominator is the number who tested positive

### Respiratory infection outbreaks

Among the total 208 sampled and tested throat swab specimens from respiratory tract infection cases during outbreak, 58 (28%) were tested positive for different influenza strains. Influenza A(H1N1)pdm2009 was responsible for the majority of the positive cases. Accordingly, from the total Influenza positive cases; 46 (79%), 10 (17%) and 2 (3%) were attributed to Influenza A(H1N1)pdm2009, A(H3N2) and Influenza B respectively (Table 3). Among the positive Influenza A(H1N1)pdm2009 cases 41% and 58% were reported in 2009 and 2010 which might be associated with 2009 pandemic influenza. More than 80% of the positive cases were among age group 15–49 years which are active in travel.

**Table 3:** Influenza activity (positivity, circulating types and subtypes) among patients sampled during outbreak investigations in Ethiopia, 2009–2015

Characteristic	Samples Collected	Samples Tested	Influenza Positive	Influenza types and subtypes			
				A	A(H1N1) pdm2009	A(H3N2)	B
	*N*	*N* (%)^a^	*N* (%)^b^	*N* (%)	*N* (%)^c^	*N* (%)^c^	*N* (%)^c^
All cases	212	208 (98)	58 (28)	56 (97)	46 (79)	10 (17)	2 (3)
Year							
2009	123	121 (98)	28 (23)	27 (96)	19 (68)	8 (29)	1 (4)
2010	82	81 (99))	29 (36)	28 (97)	27 (93)	1 (3)	1 (3)
2011	2	2 (100)	1 (50)	1 (100)	0 (0)	1 (100)	0 (0)
2012	5	4 (80)	0 (0)	0 (0)	0 (0)	0 (0)	0 (0)
2013	–	–	–	–	–	–	–
2014	–	–	–	–	–	–	–
2015	–	–	–	–	–	–	–
Age							
< 2	8	6 (75)	0 (0)	0 (0)	0 (0)	0 (0)	0 (0)
2–4	5	5 (100)	1 (20)	1 (100)	1 (100)	0 (0)	0 (0)
5–14	25	25 (100)	7 (28)	7 (100)	7 (100)	0 (0)	0 (0)
15–49	150	149 (99)	47 (32)	45 (96)	35 (74)	10 (21)	2 (4)
50–64	15	14 (93)	2 (14)	2 (100)	2 (100)	0 (0)	0 (0)
> =65	4	4 (100)	1 (25)	1 (100)	1 (100)	0 (0)	0 (0)
Missed	5	5 (100)	0 (0)	0 (0)	0 (0)	0 (0)	0 (0)
Sex							
Female	109	107 (98)	30 (28)	30 (100)	26 (87)	4 (13)	0 (0)
Male	103	101 (98)	28 (28)	26 (93)	20 (71)	6 (22)	2 (7)
Site							
Nedjo	5	4 (80)	0 (0)	0 (0)	0 (0)	0 (0)	0 (0)
Arsi	4	4 (100)	0 (0)	0 (0)	0 (0)	0 (0)	0 (0)
AU	3	2 (67)	0 (0)	0 (0)	0 (0)	0 (0)	0 (0)
Bole Air Port	3	3 (100)	0 (0)	0 (0)	0 (0)	0 (0)	0 (0)
ECA	72	71 (99)	15 (21)	14 (93)	11 (73)	3 (20)	1 (7)
Gonder	24	24 (100)	8 (33)	8 (100)	8 (100)	0 (0)	0 (0)
Metu	22	21 (95)	16 (76)	16 (100)	16 (100)	0 (0)	0 (0)
St. Paul	29	29 (100)	6 (21)	6 (100)	5 (83)	1 (17)	0 (0)
St. Peter	50	50 (100)	13 (26)	12 (92)	6 (46)	6 (46)	1 (8)

^a^Denominator is the number who were sampled

^b^Denominator is the number who were tested

^c^Denominator is the number who tested positive

### Seasonal pattern

**Fig. 2:**
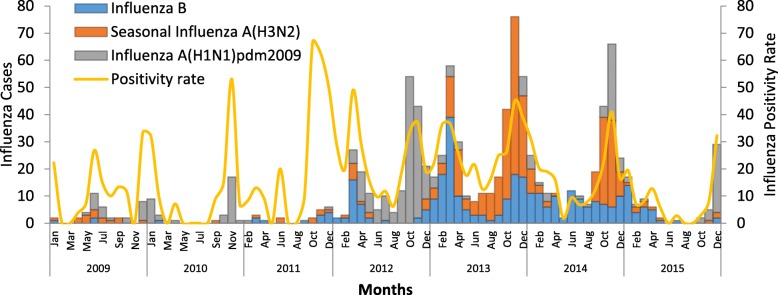
Trends of Influenza types, subtypes and proportion of positive specimens collected from ILI, SARI and outbreak sites by month per year, 2009–2015, Ethiopia

**Fig. 3:**
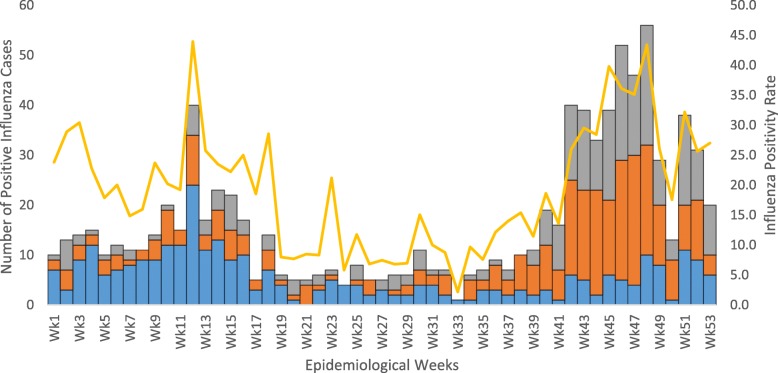
Trends of Influenza subtypes and positivity rate of specimens collected from ILI, SARI and outbreak sites by week, 2012–2015, Ethiopia

We observed positive influenza cases each month with variations in the positivity rate. The highest positivity rate was observed in November (37.5%) followed by March (27.6%), December (26.4%), October (24.4%) and January (24.3%). The lowest influenza positivity rate was observed in August (7.7%) (Table 4). The seven-year aggregated data showed that both the influenza positivity rate and the number of samples collected from the surveillance were highest in November and December. Influenza A (H1N1)pdm2009 showed a relative increase in October and November with a peak in November every year (Figs. 2, 3).

**Table 4:** Throat swab specimens collected from ILI and SARI sites and from outbreak areas and influenza positivity rate by month from 2009 to 2015, Ethiopia

Characteristic		Jan	Feb	Mar	Apr	May	Jun	Jul	Aug	Sep	Oct	Nov	Dec	Total
Samples collected	N	252	258	382	297	274	273	357	294	320	580	509	485	4281
	%	5.9	6.0	8.9	6.9	6.4	6.4	8.3	6.9	7.5	13.5	11.9	11.3	100.0
Samples Tested	N	251	247	381	291	273	269	351	286	317	579	506	484	4235
	%	5.9	5.8	9.0	6.9	6.4	6.4	8.3	6.8	7.5	13.7	11.9	11.4	100.0
Influenza Positive samples	N	61	50	105	66	25	25	32	22	48	141	190	128	893
	%	6.8	5.6	11.8	7.4	2.8	2.8	3.6	2.5	5.4	15.8	21.3	14.3	100.0
	Pos. %	24.3	20.2	27.6	22.7	9.2	9.3	9.1	7.7	15.1	24.4	37.5	26.4	21.1
Influenza A	N	25	15	38	34	15	10	18	15	37	125	164	94	590
	%	4.2	2.5	6.4	5.8	2.5	1.7	3.1	2.5	6.3	21.2	27.8	15.9	100.0
A(H1N1) pdm2009	N	11	5	13	14	8	5	10	5	12	60	73	49	265
	%	4.2	1.9	4.9	5.3	3.0	1.9	3.8	1.9	4.5	22.6	27.5	18.5	100.0
A(H3N2)	N	14	10	25	20	7	5	8	10	25	65	91	45	325
	%	4.3	3.1	7.7	6.2	2.2	1.5	2.5	3.1	7.7	20.0	28.0	13.8	100.0
Influenza B	N	36	35	67	32	10	15	14	7	11	16	26	34	303
	%	11.9	11.6	22.1	10.6	3.3	5.0	4.6	2.3	3.6	5.3	8.6	11.2	100.0

## Discussion

In this article we have presented seven years (2009–2015) of virological and epidemiological data from influenza sentinel surveillance in Ethiopia as well as results from respiratory disease outbreak investigations carried out in the country over the same period. During this time 4799 samples (representing 96.7% of all samples collected) were tested. The majority of samples which were not tested were obtained at the very start of the surveillance activities. Due to lack of viral transport media at the time, these early samples were being collected in absolute ethanol. Samples had mostly evaporated before any routine testing could begin. A total of 988 influenza positive samples were found over the seven-year period, giving a total influenza positivity rate of 20.6%. The findings indicated that seasonal Influenza A(H3N2), Pandemic Influenza A(H1N1) and Influenza B viruses were circulating in Ethiopia. Seasonal influenza A (H3N2) accounted for more than one-third (35.3%) of all influenza positive cases, followed by Influenza A(H1N1)pdm2009 (32.5%) and influenza B (32.2%).

The data from 2012 to 2015 indicated that influenza positive cases and positivity rate were predominantly identified in November (190 cases, 37.5%). Seasonal influenza A(H3N2) and Influenza A(H1N1)pdm2009 peaked in the period from September through January, with November being the month with the highest number of influenza positive cases. Similar transmission patterns have been reported for seasonal influenza A (H3N2) and influenza A (H1N1)pdm2009 viruses, supporting the idea that the spread of the Influenza A (H1N1)pdm2009 virus was similar to that of seasonal influenza A viruses [21]. However influenza B was predominantly identified in the period from November to April with a peak (67 cases, 22.1%) during March.

The influenza positivity rate was highest from September to February, mainly in November and December which are cool and dry months in Ethiopia [20, 22]. Since the data presented is predominantly from Addis Ababa (Fig. 1), the type of weather conditions described (cool and dry) is applicable for this locality during these months. The months from September to November normally correspond to the autumn season in temperate areas of the northern hemisphere. In such places the late autumn season coincides with when there is increased circulation of seasonal influenza viruses [2].

We found that the crude influenza positivity rate among ILI patients was 26.8% (21.9%, 28.7% and 25.3% for Akaki, Kolfe and Shiromeda health centers respectively). Compared to the influenza positivity rate from ILI samples in Niger (12%) and Gabon (11.4%), the influenza positivity rate among ILI samples was high in Ethiopia [23, 24]. It was very similar to the findings in Kenya, 26.7% [25] but lower than in Rwanda, 29.2% [26]. The influenza positivity rate for SARI samples was 3.1% which was low compared to findings in South Africa and China which reported 8% and 6% respectively [27, 28]. Our data showed that relatively the influenza positivity rate (27.9%) from outbreak sites was high.

The finding from ILI sentinel sites revealed that influenza positivity rate was high among children aged 5–14 years (33.2%) followed by individuals 15–44 years (26.8%) and among those older than 44 years (21.9%). Amongst SARI patients the influenza positive cases were very few, only 39 (3.1%). The majority of influenza positive cases from the SARI surveillance were identified among under-five children. Since there is no routine screening of other respiratory pathogens in either the ILI or SARI surveillance, it is not clear if the low influenza positivity rate in SARI cases might mean that most cases are being caused by pathogens other than influenza. Conversely it may indicate that the quality of SARI surveillance in Ethiopia might need significant improvement.

The primary limitation and challenges experienced by the Ethiopian Influenza sentinel surveillance system were the weak follow up of influenza-positive cases to determine the outcome of infection with influenza virus. The surveillance system has also enrolled only few SARI patients whereas the proportion of SARI as compared with total admission cases was high in all hospitals. The surveillance system is also capturing only influenza.

## Conclusion

In Ethiopia, seasonal Influenza A (H3N2), Influenza A (H1N1)pdm2009 and Influenza B are circulating. Both influenza positivity rate and number of presenting cases were predominantly observed in November showing a distinct seasonality. The surveillance has also revealed that influenza A (H3N2) and Influenza A (H1N1)pdm2009 were predominantly circulating from September through January and peaked in November while influenza B was predominantly detected from November to April with a peak in March. Hence, influenza is found one of the public health problems in Ethiopia. Introduction of vaccine and influenza antivirus is important to prevent and treat infected cases for future.

Quality influenza surveillance systems are needed to enable countries to better understand influenza epidemiology, including disease incidence and severity, and help them implement appropriate prevention strategies. A system has to be established to make a follow up on SARI cases which turned out to be positive for influenza so as to be able to determine outcome of influenza infections in all SARI cases. We also propose the introduction of laboratory diagnosis of other respiratory pathogens, especially on influenza negative samples so as to determine the proportion of other respiratory pathogens causing ILI or SARI. Finally, we recommend the inclusion of more influenza sentinel sites from different geographical and ecological areas of the country to generate more data which would be more representative of the influenza virus activity across the country so that more appropriate and comprehensive influenza control strategies are designed and implemented.

## Abbreviations

CDC: Center for Disease Control and Prevention
EPHI: Ethiopian Public Health Institute
GIS: Geographic Information System
HC: Health Center
ILI: Influenza like Illness
IQR: Interquartile range
IRR: Influenza Reagent Resource
NIL: National Influenza Laboratory
PCR: Polymerase Chain Reaction
RNA: Ribonucleic acid
RT-PCR: Real Time Polymerase Chain Reaction
SARI: Severe Acute Respiratory Infection
VTM: Viral Transport Media
WHO: World Health Organization
